# Malleability of time through progress bars and throbbers

**DOI:** 10.1038/s41598-022-14649-1

**Published:** 2022-06-21

**Authors:** Mounia Ziat, Wafa Saoud, Sonja Prychitko, Philip Servos, Simon Grondin

**Affiliations:** 1 Information Design and Corporate Communication, Bentley University, Waltham, MA 02452 USA; 2 IWK Health Centre, 5980 University Ave 5850, Halifax, NS B3K 6R8 Canada; 3 Arizona State University, 1151 S Forest Ave, Tempe, AZ 85281 USA; 4 Department of Psychology, Wilfrid Laurier University, Waterloo, ON N2L 3C5 Canada; 5 École de Psychologie, Université de Laval, Quebec, QC G1V 0A6 Canada

**Keywords:** Human behaviour, Visual system

## Abstract

Compared to a stationary pattern, a moving pattern dilates the perception of time. However, when it comes to comparing only moving stimulus, the exact dilation effects are less clear. The time dilation may be attributed to either speed of motion, temporal and spatial frequency, stimulus complexity, or the number of changes in the stimulus pattern. In the present study, we used progress bars and throbbers for inducing impressions of fast and slow “apparent” motions while the speed of motion and distance covered was actually equivalent across all conditions. The results indicate that higher number of steps produced the impression of a faster progression leading to an underestimation of time, whereas a progression in large fewer steps, produced slower apparent progression, creating the illusion of dilated time. We suggest that the perception of time depends on the nature of the stimulus rather than the speed of motion or the distance covered by the stimulus.

## Introduction

In this paper, we are concerned with the subjective experience of time when participants are exposed to familiar graphical components such as progress bars and throbbers that are part of our technological lives and give an interesting intake on time malleability and the concepts of durations and events. Durations and events are extremely relevant to the perception of space constancy and a stable world^1^. Although human beings do not have sensory receptors to assess time^2^, the perception of time is stranded to the perception of events (Joyce, 2006) from which durations are implied. The distinction between durations and events is quite interesting: on a psychological level, one’s awareness of duration is related to things that happen or change in the environment that allows one to either assess the duration of an event (absolute judgment) or to compare two durations (relative events). This time assessment can be altered by several factors such as temporal frequency^3,4^, spatial frequency^5^, stimuli complexity^6,7^, the number of changes in the stimuli pattern^8–10^, or the number of changes during a given period^11^. On a phenomenological level, a filled or empty stimulus impacts the perceived time as a full duration (or interval) or several events. An empty interval is sometimes defined as nothingness^12^ or a silent duration (without stimulation) between two sensory signals; whereas an interval is considered to be filled when the onset and offset of a continuous signal delineate the beginning and end of the interval^13,14^. The nature of unfilled and filled intervals is often referred to as the “filled duration illusion” (FDI) for indicating that the more stimuli or events are presented during a given time interval, the longer this interval is perceived^15^. These stimuli could be discrete events like flashed lights or a continuous stimulus like a path motion, making it similar to the Oppel-Kundt illusion in visual perception^16^.

**Figure 1:**
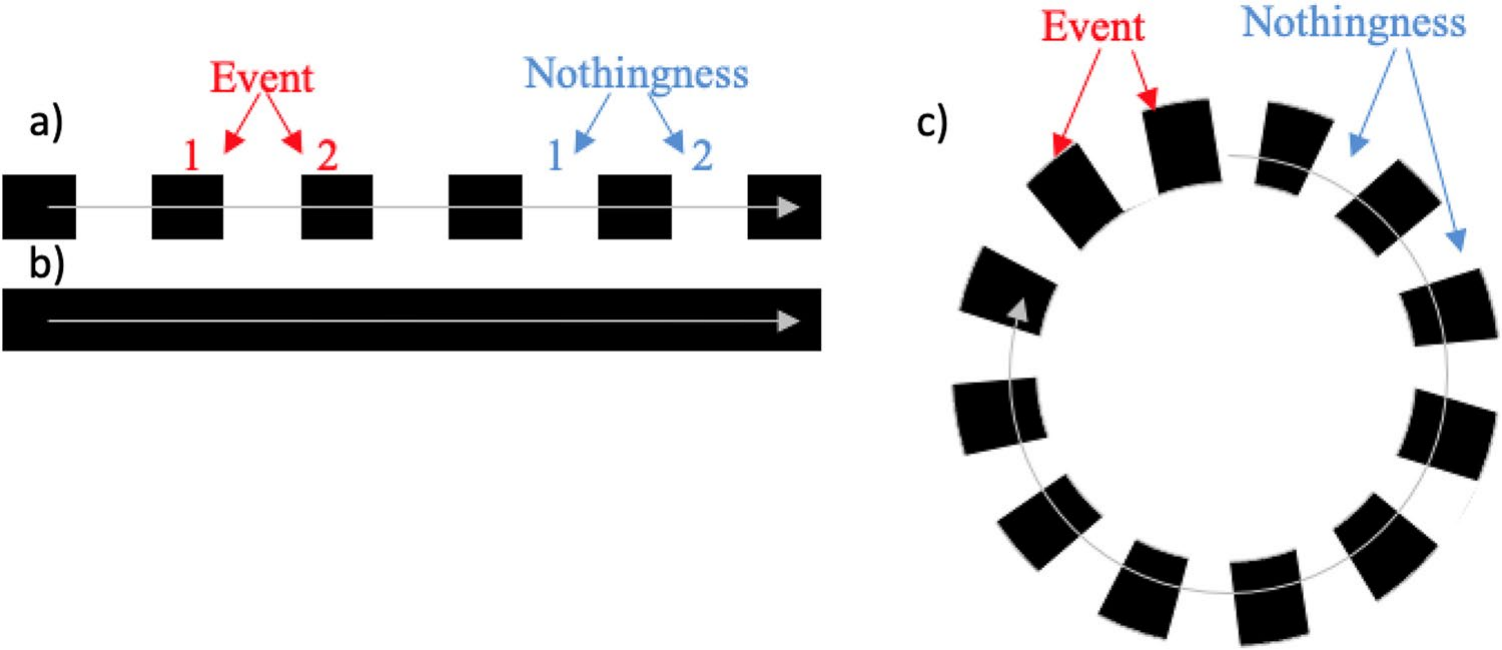
According to Bachelard, an event or an instant is something that happens between nothingness 1 and nothingness 2. By analogy, nothingness happens between two instants (i.e., linear (**a**) and rotating throbbers (**c**)). Thus, an event 1 would be the instant when the user starts the download and event 2 would be the instant when the download ends. Nothingness will be the progress that is taking place during an empty space in a throbber, which also can correspond to the concept of duration as defined by Bergson. A progress bar represents the full duration (**b**).

Whether the time stimulus is filled, or empty has an impact on the perceived duration (i.e., flashed lights vs. path motion or by analogy a throbber (Fig. 1a) vs. a progress bar (Fig. 1b)). From a perceptual perspective, the first stimulus may be experienced as a sequence, or as Bachelard’s notion of instants^12^; the second one is often experienced as a full duration as described by Bergson^17^. Bachelard refers to void time (where there is no event or stimulus) as nothingness (1). Nothingness is continuous because it is not measurable, while events or instants are discontinuous. Although nothingness is defined as empty time where there is no event or stimulus, it can be easily associated with the notion of waiting time or a silent duration. The concept of a “waiting time” is not only used in our daily lives but in most instants when one interacts with computers. In Graphical User Interface (GUI), combinations of filled and empty stimuli are often used and users’ perception of time and their tolerance in terms of “waiting time” is very important for interface design^18^ since the most common time-related interaction is related to waiting: waiting for applications to launch, downloads, updates, or for a computer to turn on or shut down. In this particular study, we are interested in progress indicators such as bars and throbbers that are often used to inform the user that an operation is in progress. Progress bars and throbbers are graphical components that are commonly used to indicate the progress of a task, such as a download or a file transfer. They give a good indication of waiting time because they often use motion to show that progress is taking place. These two graphical components are also good stimuli representative of Bergson’s duration, but they are also suitable time metaphors for Bachelard’s concept of nothingness (Fig. 1).

This distinction between duration and instants is important because they alter perceived time differently. For instance, a throbber that lasts 10 s should be perceived longer than a single progress bar of 10 s as the successive lines in a throbber might create a temporal illusion known as the kappa effect^19^, where the time interval can be biased by the spatial separation between two stimuli or two instants. Being a single stimulus that expends in time (single duration), kappa effect cannot appear with progress bars. To verify this statement, we ran a series of experiments to assess participants’ time perception when they are exposed to throbbers and progress bars that appear to move fast, slow, and constant rates. This appearance of speed changes is known as the progress bar illusion^20,21^, and although it has not been tested in throbbers, we expect a similar effect on time perception. It should be even more apparent for throbbers, i.e., a throbber moving with the same number of steps than a progress bar would be perceived faster since the number of steps will be visible to the perceiver. In the progress bar illusion, progress bars with a higher number of small steps (producing the impression of a faster bar progression) would lead to an underestimation of time, whereas a progression with a similar speed in smaller steps (produced slower apparent bar progression) with the same speed, would create the illusion of dilated time. The progress-bar illusion produces an opposite effect than the Spinner illusion^10^ whereby increasing the number of moving stimuli, the apparent speed is perceived faster than it really is. It also contradicts findings related to time perception of a moving stimuli that agree that a fast-moving stimulus dilates time and a slow-moving stimulus contracts time. Hence, a progress bar that appears to move faster should dilate time and a bar that appears to move slower should contract time.

In the present study, we investigated the relationship between time perception and stimulus motion by keeping both the distance covered by the stimuli and the speed of motion constant across all trials using progress bars and throbbers. If differences in time estimates are found, they will not be attributed to the speed or the distance. The study consists of four different experiments: Experiments 1 and 2 stimuli consisted of progress bars and throbbers respectively with shorter durations (3, 4, and 5 s); while Experiments 3 and 4 stimuli tested progress bars and throbbers respectively with longer times (10, 12, 14 s).

## Results

### Experiment 1

#### Probability of responding correctly

**Figure 2:**
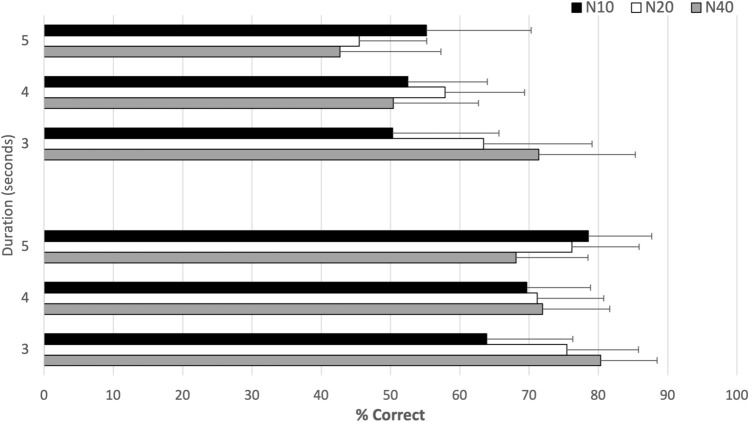
Percentage of correct responses as a function of number of steps (*N10*, *N20*, *N40*) for the three duration conditions (3s, 4s, and 5s) for Experiment 1 (progress bars) and Experiment 2 (throbbers). Errors bars show CI at 95%.

The probability of responding correctly in each experimental condition of Experiment 1 is reported in Fig. 2. This figure shows the mean correct responses for three motion rates and for three duration conditions (3, 4 and 5 s) with confidence intervals at 95%. The ANOVA revealed a significant main effect of the factor *Step*; ${F(2, 38) = 3.51, p}<$ 0.04, $\eta _p^2= 0.16$. Contrasts revealed that participants’ performances for *N40* were significantly lower, than *N20* rate. The *Step* $\times$ *Duration* interaction was also significant; ${F(4, 76) = 22.81, p}<$ 0.001, $\eta _p^2= 0.55$. This indicates that *Duration* had different effects on participants’ performances depending on which number of steps was used. To break down this interaction, we conducted simple main effect tests on each subset of the data (see Supplementary Materials). The *N40* condition at 3-s was significantly different from *N20* and *N10* conditions and the results for *N10* at 5-s were significantly different from *N20* and *N40* conditions (*p* < 0.05).

#### Error rate

**Figure 3:**
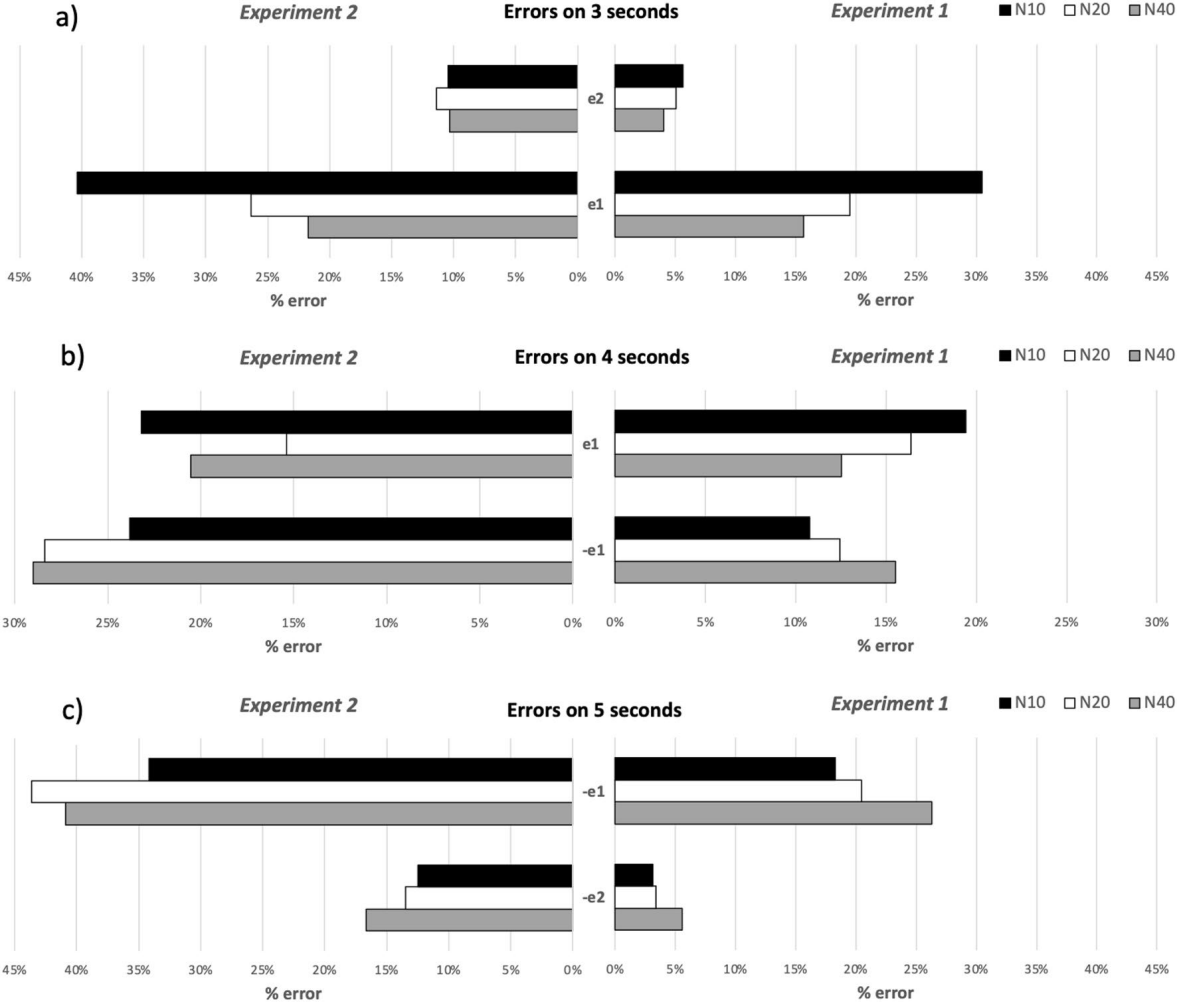
Clustered bars showing error rates at 3-s (upper panels), 4 s (middle panel) and 5 s (upper panel) per steps (*N10*, *N20*, and *N40*) and error type (− e1/2: underestimation of 1 or 2 s, e1/2: overestimation of 1 or 2 s) for Experiments 1 and 2.

In order to understand participants’ responses, we analyzed the error rate. The error, that can be either 1 s (for all three duration conditions) or 2 s (in the 3- and 5-s conditions), differs according to the number of increments in the progress bar. The three-way ANOVA on error rate at 3-s showed significant effects of the main factors *Error*; ${F(1, 19) = 41.74, p}$ < 0.001, $\eta _p^2= 0.69$, and *Step*; ${F(2, 38) = 28.32, p}$ < 0.001, $\eta _p^2= 0.60$. There was also a significant interaction between *Step* and *Error*; ${F(2, 38) = 23.99, p}$ < 0.001, $\eta _p^2= 0.56$. To break down the interaction, simple effect tests using one-way ANOVA were conducted on each subset of data. The results showed that *N40* was significantly different from *N20* and *N10* for error of 1 s (e1). Participants overestimate time mostly by 1 s, particularly when the steps condition was *N20* or *N10* and less often when the condition *N40* was presented (Fig. 3a).

At 4-s, the results showed that the interaction *Error* $\times$ *Step* was significant; ${F(2, 38) = 9.78, p}$ < 0.001, $\eta _p^2= 0.34$. No other significant effects were found. Simple main effect analysis showed that the *Step* factor has a significant effect on an overestimation of 1 s (e1) for *N10* compared to *N40*. Indeed, Fig. 3b shows that the participants made more errors when *N10* was presented.

Finally, the three-way ANOVA showed an effect of the *Error* factor, ${F(1, 19) = 34.21, p}$ < 0.001, $\eta _p^2= 0.64$, at 5-s. Figure 3c shows that − e2 error was significantly lower than error − e1 for all the three conditions. A significant effect of the *Step* factor, ${F(2, 38) = 12.73, p}$ < 0.001, $\eta _p^2= 0.40$, was also found. Post-hoc tests revealed that *N40* was significantly different from *N20* and *N10* (all pairwise comparisons can be found in Supplementary Materials). Figure 3c shows that participants had the tendency to underestimate time by 1 s when there are more steps.

### Experiment 2

#### Probability of responding correctly

Figure 2 shows the mean correct responses for three steps conditions and three duration conditions (3, 4 and 5 s). The three-way ANOVA presented a significant effect of the factor *Duration*; ${F(2,20) = 3.79, p = 0.04,}$ $\eta _p^2= 0.27$, the factor *Step*; ${F(2,20) = 10.58, p}$ < 0.05, and an interaction effect between *Duration* and *Step*; ${F(4,40) = 3.98, p = 0.008,}$ $\eta _p^2= 0.36$. This interaction suggests that the type of steps influenced the participants’ estimates of duration. In order to analyze the *Duration* and *Step* interaction, simple effect tests were conducted. The results revealed that performances for *N10* were significantly lower than *N20* and *N40* for 3-s duration. The probability of responding correctly for *N10* was also significantly lower than *N20* for the 4-s duration (all *p* values < 0.05—see Supplementary Materials).

#### Error rate

A three-way ANOVA, conducted on the error rate at 3-s, revealed a significant effect of the main factors *Error*; ${F(1, 10) = 61.58, p}$ < 0.001, $\eta _p^2= 0.86$, and *Step*, ${F(2, 20) = 8.88, p}$ < 0.05, $\eta _p^2= 0.46$. There was also a significant interaction between *Error* and *Step*; ${F(2,20) = 7.04, p}$ < 0.005, $\eta _p^2= 0.42$. Simple effects test was conducted on each subset of data to break down this interaction. The analysis revealed that the *N10* condition was significantly different from the *N20* and *N40* conditions for e1; which suggests that when the throbber is presented at fewer larger steps participants tend to overestimate time by 1 s more than in the *N20* and *N40* conditions (Fig. 3a).

At 4-s, results from the three-way ANOVA showed non-significant effect of *Duration* or *Step*. This suggests that participants’ error rates were similar across all conditions (confidence interval 16–32%) (Fig. 3b). At 5-s, results from the three-way ANOVA only revealed a significant effect of the main factor *Error*; ${F(1, 10) = 36.30, p}$ < 0.001, $\eta _p^2= 0.782$. The errors of e1 and e2 were significant, revealing the participant’s tendency to underestimate time by 1 or 2 s regardless of the number of steps (Fig. 3c). This implies that the participants would make more errors estimating a 5-s duration as a 3 or 4 s interval.

### Experiment 3

#### Probability of responding correctly

**Figure 4:**
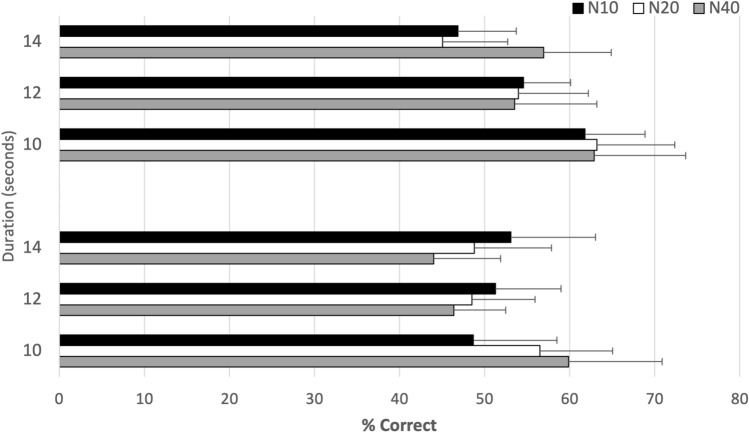
Percentage of correct responses as a function of steps (*N10*, *N20*, *N40*) for the three duration conditions (10 s, 12 s, and 14 s) for Experiment 3 (progress bars) and Experiment 4 (throbbers). Errors bars show CI at 95%.

Three-way ANOVA showed a significant effect of the interaction between *Step* and *Duration*; ${F(4, 80) = 2.53, p}$ < 0.05, $\eta _p^2= 0.11$. To break down this interaction, simple pairwise comparisons were performed. The results showed that *N10* was significantly different from *N40* for the 10-s condition (see Supplementary Materials). Figure 4 shows that the probability of responding correctly is higher when participants are presented with more steps (*N40*) for the 10-s duration.

#### Error rate

**Figure 5:**
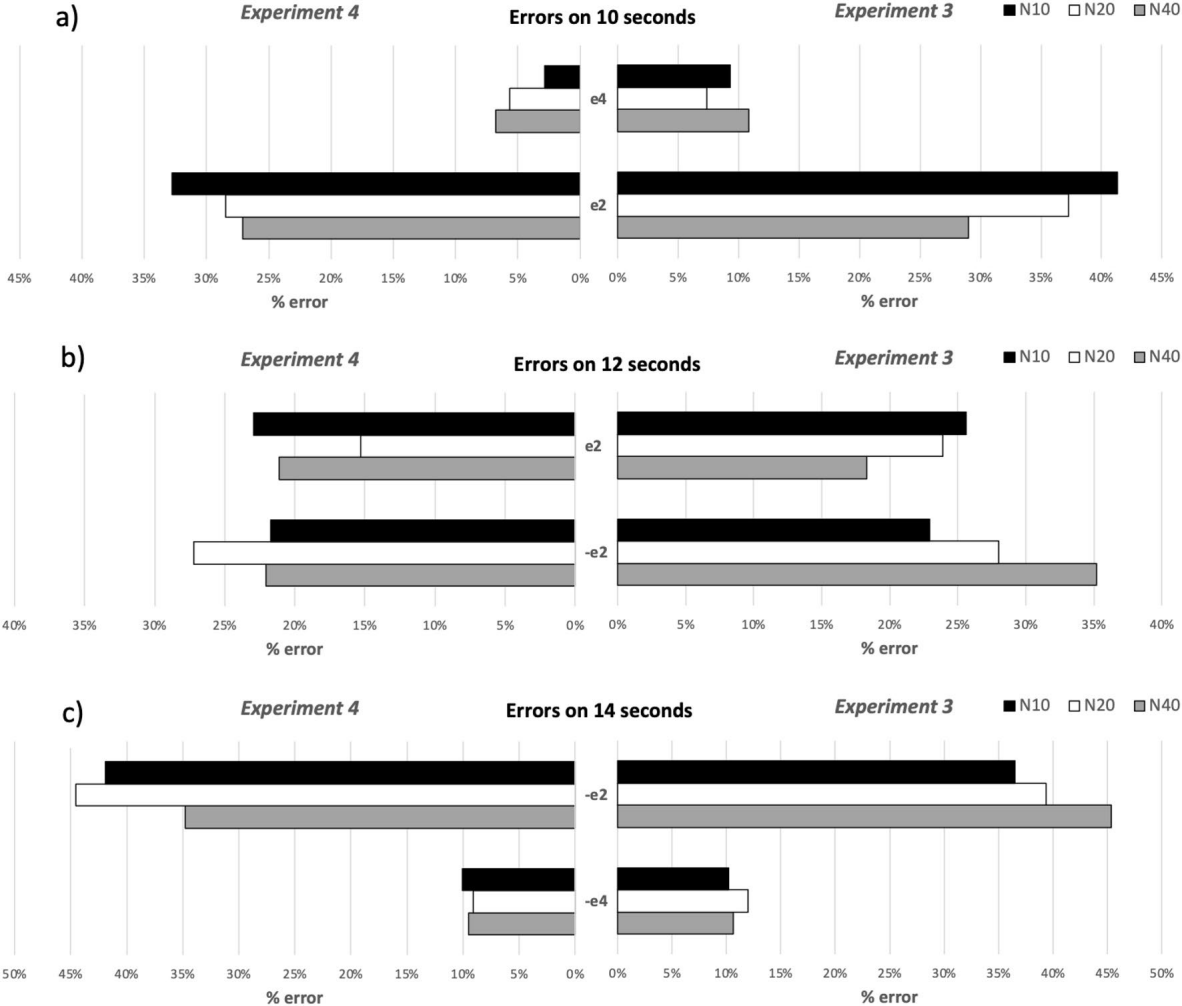
Clustered bars showing error rates at 10-s (upper panels), 12 s (middle panel) and 14 s (upper panel) per steps (*N10*, *N20*, and *N40*) and error type (− e2/4: underestimation of 2 or 4 s, e2/4: overestimation of 2 or 4 s)—experiments 3 and 4.

The three-way ANOVA on error rate for duration 10 showed significant effects of the main factors *Error*; ${F(1, 20) = 72.40, p}$ < 0.001, $\eta _p^2= 0.78$, and *Step*; ${F(2, 40) = 4.32, p}$ < 0.05, $\eta _p^2= 0.18$. There was also a significant interaction between *Step* and *Error*; ${F(2, 40) = 4.80, p}$ < 0.05, $\eta _p^2= 0.19$. To break down the interaction, simple effect tests were conducted on each subset of data (details in Supplementary Materials). The results showed significant differences between *N40* and *N20* and between *N40* and *N10* for error e2. Figure 5a shows that the participants’ error e2 was significantly lower comparing to *N20* and *N10* conditions. In other words, participants were more accurate when they were performing the task with the highest number of steps.

At 12-s, results showed that the *Error* and *Step* interaction was significant; ${F(2, 40) = 4.65, p}$ < 0.05, $\eta _p^2= 0.19$. No other significant effects were found. Pairwise comparisons showed that the *N40* condition was significantly different from the *N10* condition for error (− e2). Indeed, Fig. 5b shows that participants overestimated more often the duration by 2 s with the higher number of steps, while the error is reduced when it comes to lower number of steps.

Finally, the three-way ANOVA showed an effect of the *Error* factor ${F(1, 20) = 150.85, p}$ < 0.001, $\eta _p^2= 0.88$, at 14-s. Figure 5c shows that an error of 2 s (− e2) is higher than an error of 4 s, i.e., participants easily confused 12- and 14-s progress bars and distinguished the 10-s progress bar from others regardless the used number of steps.

### Experiment 4

#### Probability of responding correctly

The percentage of correct responses are displayed on Fig. 4 for the three steps and three durations. For Experiment 4, the ANOVA indicated a significant main effect of *Duration*, $${F(2, 36) = 3.65,p = 0.036,}$$$$\eta _p^2= 0.857$$, regardless the steps. Performances for the 10-s duration were significantly higher than the 12- and 14-s conditions.

#### Error rate

At 10-s, the three-way ANOVA presented a significant effect of the main factor *Error*; ${F(1, 15) = 96.26, p}$ < 0.001, $\eta _p^2= 0.862$. Errors of e1 and e2 were both significantly different from each other, which indicates that the overestimation of time was higher by 2 regardless of the number of steps (Fi. 5a). The ANOVA indicated no significant effect of *Error* at 12 s, which suggests that participants tended made equally errors of type e2 and − e2 (Fig. 5b). Finally, the ANOVA revealed a significant effect of the *Error* factor, ${F(1, 15) = 93.27, p}$ < 0.001, $\eta _p^2= 0.862$, at 14-s. The participants’ errors of e1 and e2 were significant from each other, which shows that the participants underestimated time by 2 more often, regardless of number of steps (Fig. 5c).

### Same stimulus comparison

#### Experiment 1 vs. Experiment 3

The Split-Plot ANOVA with the within-subjects factors Step, Duration, and Experiment type as between-subjects factor was followed by pairwise comparisons (see Supplementary Materials). For similar number of steps (*N10*, *N20*, *N40*), the probability of answering correctly dropped significantly. For both experiments, pairwise comparisons shows that the correct response rate was significantly higher (*p* < 0.05) when associated with *N40* for the shortest duration (3-s/10-s) and *N10* for the longest duration (5-s/14-s). When observers committed errors, the errors were due to the variation in the number of steps that occurred during the progression. For the shortest duration (3-s/10-s), participants’ error rate was significantly higher for type (e1/2) than type (e2/4) (respond more 4 than 5-s and more 12 than 14-s). The overestimation of time was significantly higher for step *N10* for both experiments; with this trend being significantly higher for Experiment 3. Additionally, for Experiment 3, a significant overestimation occurred for *N20* comparing to *N40* producing more errors. For the intermediate duration (4-s/12-s), all errors were significantly higher for type (− e1/2) than type (e1/2) leading to higher underestimation for *N40* for Experiment 3 comparing to *N20* and *N10*. Finally, for the longest duration for each set (5-s/14-s): type (− e1/2) error was significantly higher than type (− e2/4) error. There was a higher underestimation for *N40* for Experiment 3 comparing to *N10*. In sum, the lower the number of steps, the higher the overestimation of time and conversely the higher the number of steps, the higher the underestimation of time, which corroborates our initial hypothesis related to progress bars.

#### Experiment 2 vs. Experiment 4

Participants’ probability of answering correctly was similar for both bcexperiments except for the longest duration (5-s and 14-s) when associated with the highest number of steps (*N40*). There was a significant error rate for this duration (*p* < 0.05). Participants were less accurate in Experiment 2 than Experiment 4 estimating the duration when the longest duration was combined with *N20* and *N40* making more type − e1/2 errors. We observed an opposite trend for error type − e2/4 for step *N20*, where the error rate was higher for Experiment 4. Finally, for the *N10* condition, there were more type − e2/4 error in Experiment 2 than in Experiment 4. The longest duration led to a larger underestimation of time in Experiment 2 than in Experiment 4 when associated with all three steps, while the intermediate duration lead to an opposite effect with an increase in error rate for Experiment 4.

### Progress bar—throbber comparison

#### Experiment 1 vs. Experiment 2

There was no difference in performance between progress bars and throbber for the shortest duration (3-s). For the intermediate duration (4-s), there was a significant difference between progress bars and throbbers for the lowest number of steps (*N10*) and the highest number of steps (*N40*), with the probability of answering correctly dropping significantly for throbbers (*p* < 0.05). Similar results were found for the longest duration (5-s), a significant reduction in correct responses for throbbers were found for all the three steps.

Error rates for the shortest duration were similar. For the intermediate duration, there was significantly more errors of type − e1 using throbbers than using the progress bars across all *Step* conditions. This trend translated in a higher error rates for throbbers that were significant at 4-s for error type − e1 for the three steps conditions: *N10*, *N20*, and *N40* (Fig. 3b,c). At 5-s for both error types − e1 and − e2, performances for throbbers significantly dropped for the three steps *N10*, *N20*, and *N40* for all three durations.

#### Experiment 3 vs. Experiment 4

We observed an opposite trend for longer durations (10, 12, and 14). Performances were significantly better for throbbers than for progress bars at 10-s for the *N10* condition and at 14-s for the *N40* condition (*p* < 0.05). Finally, participants made more errors for throbbers by underestimating time by 2 s using progress bars with both the intermediate duration (12 s) and the longest duration (14 s) when combined with *N40*.

## Discussion

Experiments 1 and 3 showed that when the progress bar contains higher number of steps, time tends to move faster and when the progress bar contains fewer number of steps, time tends to be dilated. For both experiments, our results showed that participants tend to overestimate time for the lower number of steps when associated with the shortest durations (3-s/10-s) and they tend to underestimate time for higher number of steps when associated with the longest durations (5-s/14-s). The intermediate duration led to an overestimation of time but only for Experiment 3. Experiments 2 and 4, using throbbers shows similar trends than progress bars with higher steps leading to a larger time contraction. For both Experiments 2 and 4, regardless the number of steps, the longest duration associated with the highest number of steps led to a larger underestimation of time. The overestimation and underestimation errors were equal for Experiment 4. These results validate Harrison et al.’s concept of “progress bar illusion”^20,22^. For Experiments 1 and 2, participants were less accurate estimating the correct duration when they were exposed to throbbers as opposed to progress bars, leading to a larger underestimation of time for intermediate and longest durations for throbbers. Interestingly, when comparing Experiment 3 to Experiment 4, for the shortest periods (10-s), time estimation with a stimulus containing nothingness or silent durations moving with fewer steps lead to more accurate results as compared to a filled stimulus. For intermediate and longest durations, participants underestimated time more often using progress bars than throbbers when using the highest number of steps (*N40*).

Our results seem to diverge from the findings in the literature that show that rapid moving visual stimuli either dilate time perception or have no effect^23–27^. The effect is even more prominent for throbbers than progress bars and contradict the results of the FDI, wherein the more stimuli or events during a given time interval, the higher the dilation of time. One can argue that those contradicting findings are due to the fact that moving fast or slow is purely an optical illusion. All stimuli are of the same distance and speed, with the only variable factors are the inclusion of nothingness durations and the number of events. Changing the number of steps only creates the illusion of a faster motion because the number of steps has been increased. Therefore, if the speed and distance remains the same, increasing the number of events should lead to an underestimation of time. Interestingly, researchers showed that speed of motion is not responsible for time dilation and that the overestimation of time is attributed to the temporal frequency^3,4^. It is worth mentioning that, most of studies cited above used a temporal reproduction task rather than a temporal judgment task. Matthews^21^ showed that temporal reproduction tasks are mainly based on remembering duration which corresponds to the subject passage of time during the reproduction interval and pointed out the importance of the nature of the stimulus used for estimating duration.

One plausible explanation comes from a study of Orgs et al.^28^ who tested the effect of apparent motion on time perception. They showed that participants experienced an apparent biological motion in two different paths (short and long) by changing the order of the pictures showing body postures. The “long sequence” that produced an apparent motion that was about one and a half times longer than those in the “short sequence”, was perceived as shorter. We would expect that the longer sequence would produce a time dilation, because they would take longer to execute. However, the opposite was found and the long sequences were perceived to take less time than the short sequences, confirming our findings that the subjective experience of the durations had been compressed. Similarly to the progress bar illusion, manipulating the apparent motion produced directly proportional changes in the perceived speed of the movement and inversely proportional changes in time perception. So, sequences that imply longer movement paths produced faster apparent motion and a compression of subjective time, whereas sequences that imply shorter paths produced slower apparent motion and dilated the sequence duration. Similarly, quick higher steps produced faster apparent progression of the bar or the throbber and led to a compression of time, whereas large fewer steps produced slower apparent progression and dilated time perception. The fact that this effect was preeminent with throbbers was accentuated by the silent durations that potentially indicated a faster progression.

Additionally, the nature of the stimuli itself and the representation users make of it could have play a role. Our universal knowledge of progress bars and throbbers is associated with the concept of “waiting time” and although we did not mentioned this information to participants, it could have affected their judgment. This bring us to the initial philosophical debate between Bachelard and Bergson about both the elasticity and fleetingness of time, but also the notable public debate between Einstein and Bergson in Paris in 1922 between the definition of time in quantum physics and phenomenology^29^. Our subjective experience of time and the temporal information comes not only from the visual sense, but also from the other sensory modalities such as touch and audition, which also exhibit special effects of space on time perception^30–34^. Not to mention the effects of emotions and social context on time perception^35–38^.

As a continuation of this work, we are planning to use the progress bar illusion on vertical and circular paths and evaluate the effect of the other modalities on this paradigm. By using an empty interval paradigm, Ono and Kitazawa^39^ showed that the duration expending stimulus is perceived as shorter while the duration of a receding stimulus is perceived as longer. They concluded that the time contraction is caused by collision anticipation. Circular paths are of a particular interest because, in this case, it will be associated with a zoom paradigm (an object expending or receding)^40,41^ and will offer an ideal condition for using filled intervals.

## Methods

### Experiment 1

#### Participants

Twenty students (mean age, 21.0 ± 1.3 years; 12 females) of Wilfrid Laurier University participated in exchange for compensation in the experiment. All participants provided written informed consent for the procedures in this study. They had normal or corrected-to-normal refraction and normal visual acuity. Wilfrid Laurier University Research Ethics Board (REB) approved the experimental procedures and the experiment was performed in accordance with relevant guidelines and regulations.

#### Stimuli and apparatus

The stimuli were created on a MAC running Leopard using the Psychophysics Toolbox extensions (Brainard, 1997; Pelli, 1997) running on MATLAB 2009 (MathWorks, Nantucket, Massachusetts). The stimulus consisted of a progress bar moving for a duration of 3, 4 or 5 s. For each duration, the number of steps during the progression was changed, creating the illusion of a bar moving slower or faster^20^. The intermediate condition *N20* corresponds to a progress bar moving linearly at 20 steps. An apparent slow progression consisted of 10 large steps (*N10*) and an apparent fast progression consisted of 40 increments (*N40*). Harrison et al.^22^ showed that when steps become more frequent, it creates the illusion of faster motion and reduces the perceived duration by 11%. Note that in the three situations the speed of motion and the distance traveled remains the same. Moving constantly, faster, or slower is purely an optical illusion created by the number of steps that are not visible to the perceiver in a progress bar. The bar started to progress from either the right or the left. The progress bar was 440 $\times$ 50 pixels, in a blue color (RGB: 0-0-255) and was displayed on the center of a 27 in. screen (iMac) with a black background. The monitor had a resolution of 1920 $\times$ 1200 pixels with vertical frame rate of 60 Hz and a gamma of 1.8 and was observed binocularly from a distance of 83 cm, resulting in 38 pixels per degree of visual angle. The experiments were carried out in a dark room except for the dim light behind the display that provided partial light adaptation to reduce visual persistence of objects on the screen.

#### Procedure and task

The experimental design was completely symmetrical and counterbalanced. It comprised two blocks of 270 trials. In each block, a randomized mix of three durations (3, 4 or 5s), either *N20*, *N10*, or *N40* progress was presented in equal proportions. The motion direction of the progress bar, left or right was also randomized in equal proportions. Each trial started with a fixation white dot displayed at the center of the screen. The dot disappeared before the presentation of the stimulus. A sound was played after the cessation of the progress bar to inform the participant to give the answer by pressing on one of the keys marked 3, 4 or 5. A new trial was initiated only after the participant’s response, thus the experiment proceeded at a pace determined by the participant. The participants were specifically instructed to refrain from using an explicit counting strategy.

### Experiment 2

#### Participants

Eleven students (mean age 20.7 ± 3.0 years; eight females) of Northern Michigan University voluntarily participated in Experiment 2. They all read and signed an informed consent statement about the procedures in this study and had normal or corrected vision and normal visual acuity. The Institutional Review Board (IRB) of Northern Michigan University approved the experimental procedures and the experiment was performed in accordance with relevant guidelines and regulations.

#### Stimuli and apparatus

The apparatus used in this second experiment was similar to the one used in Experiment 1. The stimulus was a throbber moving for a duration of 3, 4, or 5 s. For each duration, the number of steps during the progression of the throbber were one of the three following conditions: The *N20* condition consisted of a throbber moving at 20 steps: with 10 filled steps and 10 empty steps. The *N10* condition involved a progression of 10 large steps (5 filled steps and 5 empty steps), and the *N40* condition was comprised of a progression of 40 increments with a quick apparent motion (20 filled steps and 20 empty steps). This creates a spinner-like illusion^10^ with one difference between our stimuli and those in spinner illusion resides in the empty/filled ratio. The ratio is kept at 1 throughout all experiment conditions, with the width of empty/filled remaining the same. In a spinner illusion, the empty space is not necessarily equal to the filled space. The throbber started on either the right or the left of the computer screen. The throbber was similar to the size of the progress bar and was 440 $\times$ 50 pixels in a blue color (RGB: 0-0-255) for the filled steps and black for the empty steps. It was displayed on the center of a black background on a 27-in. screen.

#### Procedure and task

The procedure is similar to Experiment 1 and the experimental design was symmetrical and counterbalanced and comprised two blocks of 150 trials. During each block of trials, the three durations (3, 4, or 5s) and progress steps of *N10*, *N20*, or *N40* were randomized and presented in equal proportions. The motion direction (left or right) of the throbber was also randomized in equal proportions. Participants gave an answer by pressing one of the keys marked 3, 4, or 5.

### Experiment 3

#### Participants

Twenty-one students (mean age, 21.0 ± 1.2 years; 12 females) of Wilfrid Laurier University participated in exchange for compensation in this third experiment. All participants provided written informed consent for the procedures in this study. They had normal or corrected-to-normal refraction and normal visual acuity. Wilfrid Laurier University REB approved the experimental procedures and the experiment was performed in accordance with relevant guidelines and regulations.

#### Stimuli and apparatus

The stimuli and apparatus used in this third experiment were similar to the one used in Experiment 1. The main difference resided in the fact that the progress bar durations were 10, 12 or 14 s. The number of steps during the progression were similar to Experiment 1 with a *N20* condition (20 steps), a *N10* condition (10 steps), and a *N40* condition (40 steps).

#### Procedure and task

The experimental design was completely symmetrical and counterbalanced and it comprised two blocks of 90 trials with a randomized mix of three durations (10, 12, 14s) and the three steps (*N10*, *N20*, or *N40*) were presented in equal proportions. The participant to give the answer by pressing on one of the keys marked 10, 12, 14.

### Experiment 4

#### Participants

Sixteen students (mean age 19.3 ± 1.3 years; nine females) from Northern Michigan University participated in Experiment 4. Before the experiment, they read and signed the informed consent that has been approved by the Institutional Review Board of Northern Michigan University along with the experimental procedures. The experiment was performed in accordance with relevant guidelines and regulations.

#### Stimuli and apparatus

The stimuli and apparatus used in Experiment 4 were similar to those of Experiment 3. In this fourth experiment, the stimulus was a throbber moving for a duration of 10, 12, or 14 s.

#### Procedure and task

The procedure and task were similarly to the previous experiments. Experiment 4 comprised two blocks of 90 trials and participants were asked to give an answer by pressing one of the keys marked 10, 12, or 14.

### Data analysis

A 2 (direction) $\times$ 3 (duration) $\times$ 3 (steps) repeated-measures Analysis of Variance (ANOVA) was conducted on the participants’ correct responses. Simple Main Effects were conducted when interaction was significant. Pairwise comparisons were used to examine the difference of the main effects. As for the error rates, they were analyzed separately for each duration with a 2 (direction) $\times$ 2 (error) $\times$ 3 (steps) with a repeated-measures ANOVA. Because there was no effect of the direction, the analysis did not include Direction as a factor. Greenhouse–Geisser corrections were used when the sphericity assumption was violated. Significance level was set to 0.05 and post-hoc tests were performed using paired sampled t-tests with a Bonferroni correction. Only significant results were presented. For between-experiments comparison, we opted for a Split-Plot ANOVA with Step, Duration as within-subjects factors and Experiment type as between-subjects factor. It was was followed by pairwise comparisons of the main effects.

## Supplementary Information

Supplementary Information.

## Data Availability

The datasets generated during the current study are available from the corresponding author on reasonable request.
