# Supplementary material for: Malleability of time through progress bars and throbbers
Source: Sci Rep. 2022 Jun 21;12:10400. doi: 10.1038/s41598-022-14649-1 (PMC9213475; doi:10.1038/s41598-022-14649-1)
Supplement: Supplementary file 1 — Supplementary Information. [file 41598_2022_14649_MOESM1_ESM.pdf]

SUPPLEMENTARY MATERIALS

MALLEABILITY OF TIME THROUGH PROGRESS BARS AND THROBBERS

PAIRWISE COMPARISONS

EXPERIMENT 1

PROBABILITY OF RESPONDING CORRECTLY

DURATION (1:SHORT (3S), 2:INTERMEDIATE (4S), 3:LONG (5S)) * STEPS (1:N40, 2:N20, 3:N10)

Pairwise Comparisons

Measure: MEASURE_1

Duration	(I) Steps	(J) Steps	Mean Difference (I−J)	Std. Error	Sig.[b]	95% Confidence Interval for Difference[b]	
						Lower Bound	Upper Bound
1	1	2	-11.559*	1.854	<.001	-16.427	-6.692
		3	-16.426*	2.757	<.001	-23.663	-9.190
	2	1	11.559*	1.854	<.001	6.692	16.427
		3	-4.867	2.009	.077	-10.142	.408
	3	1	16.426*	2.757	<.001	9.190	23.663
		2	4.867	2.009	.077	-.408	10.142
2	1	2	-1.505	2.184	1.000	-7.239	4.230
		3	-2.264	1.977	.799	-7.454	2.925
	2	1	1.505	2.184	1.000	-4.230	7.239
		3	-.760	1.976	1.000	-5.948	4.428
	3	1	2.264	1.977	.799	-2.925	7.454
		2	.760	1.976	1.000	-4.428	5.948
3	1	2	2.373	2.201	.883	-3.406	8.153
		3	10.433*	2.499	.002	3.873	16.993
	2	1	-2.373	2.201	.883	-8.153	3.406
		3	8.060*	1.823	<.001	3.274	12.845
	3	1	-10.433*	2.499	.002	-16.993	-3.873
		2	-8.059*	1.823	<.001	-12.845	-3.274

Based on estimated marginal means

*. The mean difference is significant at the .05 level.

b. Adjustment for multiple comparisons: Bonferroni.

ERROR RATE AT 3 SEC.

ERROR (1:E1, 2: E2) * STEP (1:N40, 2:N20, 3:N10)

Pairwise Comparisons

Measure: MEASURE_1

Error	(I) Steps	(J) Steps	Mean Difference (I−J)	Std. Error	Sig.[b]	95% Confidence Interval for Difference[b]	
						Lower Bound	Upper Bound
1	1	2	.140*	.035	.007	.040	.240
		3	.185*	.046	.006	.055	.315
	2	1	-.140*	.035	.007	-.240	-.040
		3	.045	.037	.749	-.059	.149
	3	1	-.185*	.046	.006	-.315	-.055
		2	-.045	.037	.749	-.149	.059
2	1	2	-.009	.027	1.000	-.084	.066
		3	.002	.010	1.000	-.027	.030
	2	1	.009	.027	1.000	-.066	.084
		3	.011	.020	1.000	-.046	.068
	3	1	-.002	.010	1.000	-.030	.027
		2	-.011	.020	1.000	-.068	.046

Based on estimated marginal means

*. The mean difference is significant at the .05 level.

b. Adjustment for multiple comparisons: Bonferroni.

Pairwise Comparisons

Measure: MEASURE_1

Steps	(I) Error	(J) Error	Mean Difference (I−J)	Std. Error	Sig.[b]	95% Confidence Interval for Difference[b]	
						Lower Bound	Upper Bound
1	1	2	.298*	.045	<.001	.199	.396
	2	1	-.297*	.045	<.001	-.396	-.199
2	1	2	.148*	.034	.001	.073	.224
	2	1	-.148*	.034	.001	-.224	-.073
3	1	2	.114*	.022	<.001	.065	.163
	2	1	-.114*	.022	<.001	-.163	-.065

Based on estimated marginal means

*. The mean difference is significant at the .05 level.

b. Adjustment for multiple comparisons: Bonferroni.

ERROR RATE AT 4 SEC.

ERROR (1:-E1, 2: E1) * STEP (1:N40, 2:N20, 3:N10)

Pairwise Comparisons

Measure: MEASURE_1

error	(I) step	(J) step	Mean Difference (I-J)	Std. Error	Sig.[b]	95% Confidence Interval for Difference[b]	
						Lower Bound	Upper Bound
1	1	2	-1.653	1.087	.434	-4.505	1.199
		3	-4.738	2.039	.094	-10.089	.614
	2	1	1.653	1.087	.434	-1.199	4.505
		3	-3.085	1.624	.218	-7.346	1.177
	3	1	4.738	2.039	.094	-.614	10.089
		2	3.085	1.624	.218	-1.177	7.346
2	1	2	3.067	2.018	.435	-2.230	8.363
		3	6.910*	1.323	<.001	3.437	10.382
	2	1	-3.067	2.018	.435	-8.363	2.230
		3	3.843	1.727	.115	-.690	8.376
	3	1	-6.910*	1.323	<.001	-10.382	-3.437
		2	-3.843	1.727	.115	-8.376	.690

Based on estimated marginal means

*. The mean difference is significant at the .05 level.

b. Adjustment for multiple comparisons: Bonferroni.

ERROR RATE AT 5 SEC.

ERROR (1:-E1, 2: -E2) * STEP (1:N40, 2:N20, 3:N10)

Pairwise Comparisons

Measure: MEASURE_1

error	(I) step	(J) step	Mean Difference (I-J)	Std. Error	Sig.[b]	95% Confidence Interval for Difference[b]	
						Lower Bound	Upper Bound
1	1	2	-.002	.010	1.000	-.028	.025
		3	-.025	.014	.260	-.060	.011
	2	1	.002	.010	1.000	-.025	.028
		3	-.023	.011	.167	-.053	.007
	3	1	.025	.014	.260	-.011	.060
		2	.023	.011	.167	-.007	.053
2	1	2	-.022	.025	1.000	-.088	.044
		3	-.080*	.025	.016	-.147	-.013
	2	1	.022	.025	1.000	-.044	.088
		3	-.058*	.021	.033	-.112	-.004
	3	1	.080*	.025	.016	.013	.147
		2	.058*	.021	.033	.004	.112

Based on estimated marginal means

*. The mean difference is significant at the .05 level.

b. Adjustment for multiple comparisons: Bonferroni.

Pairwise Comparisons

Measure: MEASURE_1

step	(I) error	(J) error	Mean Difference (I-J)	Std. Error	Sig.[b]	95% Confidence Interval for Difference[b]	
						Lower Bound	Upper Bound
1	1	2	-.152*	.036	<.001	-.227	-.077
	2	1	.152*	.036	<.001	.077	.227
2	1	2	-.172*	.033	<.001	-.242	-.103
	2	1	.173*	.033	<.001	.103	.242
3	1	2	-.208*	.036	<.001	-.283	-.132
	2	1	.208*	.036	<.001	.132	.283

Based on estimated marginal means

*. The mean difference is significant at the .05 level.

b. Adjustment for multiple comparisons: Bonferroni.

EXPERIMENT 2

PROBABILITY OF RESPONDING CORRECTLY

DURATION (1:SHORT (3S), 2:INTERMEDIATE (4S), 3:LONG (5S)) * STEPS (1:N40, 2:N20, 3:N10)

Pairwise Comparisons

Measure: MEASURE_1

duration	(I) step	(J) step	Mean Difference (I–J)	Std. Error	Sig.[b]	95% Confidence Interval for Difference[b] Lower Bound	Upper Bound
1	1	2	-13.081*	4.435	.044	-25.808	-.354
		3	-21.059*	5.791	.014	-37.680	-4.438
	2	1	13.081*	4.435	.044	.354	25.808
		3	-7.978	5.260	.481	-23.076	7.119
	3	1	21.059*	5.791	.014	4.438	37.680
		2	7.978	5.260	.481	-7.119	23.076
2	1	2	-11.813*	4.021	.045	-23.354	-.271
		3	-9.533	3.516	.066	-19.623	.557
	2	1	11.813*	4.021	.045	.271	23.354
		3	2.280	1.997	.840	-3.451	8.011
	3	1	9.533	3.516	.066	-.557	19.623
		2	-2.280	1.997	.840	-8.011	3.451
3	1	2	-5.382	3.622	.504	-15.777	5.013
		3	2.109	4.966	1.000	-12.145	16.363
	2	1	5.382	3.622	.504	-5.013	15.777
		3	7.491	3.260	.133	-1.864	16.846
	3	1	-2.109	4.966	1.000	-16.363	12.145
		2	-7.491	3.260	.133	-16.846	1.864

Based on estimated marginal means

*. The mean difference is significant at the .05 level.

b. Adjustment for multiple comparisons: Bonferroni.

Pairwise Comparisons

Measure: MEASURE_1

step	(I) duration	(J) duration	Mean Difference (I–J)	Std. Error	Sig.[b]	95% Confidence Interval for Difference[b] Lower Bound	Upper Bound
1	1	2	1.802	5.047	1.000	-12.683	16.286
		3	-2.182	7.690	1.000	-24.254	19.890
	2	1	-1.802	5.047	1.000	-16.286	12.683
		3	-3.984	3.809	.961	-14.915	6.948
	3	1	2.182	7.690	1.000	-19.890	24.254
		2	3.984	3.809	.961	-6.948	14.915
2	1	2	3.070	2.967	.975	-5.444	11.584
		3	5.517	5.023	.893	-8.899	19.933
	2	1	-3.070	2.967	.975	-11.584	5.444
		3	2.447	2.961	1.000	-6.051	10.945
	3	1	-5.517	5.023	.893	-19.933	8.899
		2	-2.447	2.961	1.000	-10.945	6.051
3	1	2	13.328*	3.085	.005	4.474	22.183
		3	20.986*	5.508	.010	5.177	36.796
	2	1	-13.328*	3.085	.005	-22.183	-4.474
		3	7.658	3.143	.105	-1.361	16.678
	3	1	-20.986*	5.508	.010	-36.796	-5.177
		2	-7.658	3.143	.105	-16.678	1.361

Based on estimated marginal means

*. The mean difference is significant at the .05 level.

b. Adjustment for multiple comparisons: Bonferroni.

ERROR RATE AT 3 SEC.

ERROR (1:E1, 2: E2) * STEP (1:N40, 2:N20, 3:N10)

Pairwise Comparisons

Measure: MEASURE_1

error	(I) step	(J) step	Mean Difference (I–J)	Std. Error	Sig.[b]	95% Confidence Interval for Difference[b] Lower Bound	Upper Bound
1	1	2	.140*	.039	.014	.029	.251
		3	.185*	.051	.013	.040	.331
	2	1	-.140*	.039	.014	-.251	-.029
		3	.045	.041	.866	-.071	.162
	3	1	-.185*	.051	.013	-.331	-.040
		2	-.045	.041	.866	-.162	.071
2	1	2	-.009	.029	1.000	-.093	.074
		3	.002	.011	1.000	-.030	.033
	2	1	.009	.029	1.000	-.074	.093
		3	.011	.022	1.000	-.052	.074
	3	1	-.002	.011	1.000	-.033	.030
		2	-.011	.022	1.000	-.074	.052

Based on estimated marginal means

*. The mean difference is significant at the .05 level.

b. Adjustment for multiple comparisons: Bonferroni.

Pairwise Comparisons

Measure: MEASURE_1

step	(I) error	(J) error	Mean Difference (I–J)	Std. Error	Sig.[b]	95% Confidence Interval for Difference[b] Lower Bound	Upper Bound
1	1	2	.297*	.049	<.001	.188	.406
	2	1	-.297*	.049	<.001	-.406	-.188
2	1	2	.148*	.038	.003	.064	.232
	2	1	-.148*	.038	.003	-.232	-.064
3	1	2	.114*	.024	<.001	.060	.168
	2	1	-.114*	.024	<.001	-.168	-.060

Based on estimated marginal means

*. The mean difference is significant at the .05 level.

b. Adjustment for multiple comparisons: Bonferroni.

ERROR RATE AT 4 SEC.

ERROR (1:-E1, 2: E1) * STEP (1:N40, 2:N20, 3:N10)

Pairwise Comparisons

Measure: MEASURE_1

step	(I) error	(J) error	Mean Difference (I-J)	Std. Error	Sig.[b]	95% Confidence Interval for Difference[b]	
						Lower Bound	Upper Bound
1	1	2	.005	.083	.957	-.180	.189
	2	1	-.005	.083	.957	-.189	.180
2	1	2	.129*	.053	.034	.012	.246
	2	1	-.129*	.053	.034	-.246	-.012
3	1	2	.086	.077	.290	-.086	.259
	2	1	-.086	.077	.290	-.259	.086

Based on estimated marginal means

*. The mean difference is significant at the .05 level.

b. Adjustment for multiple comparisons: Bonferroni.

ERROR RATE AT 5 SEC.

ERROR (1:-E1, 2: -E2) * STEP (1:N40, 2:N20, 3:N10)

Pairwise Comparisons

Measure: MEASURE_1

step	(I) error	(J) error	Mean Difference (I-J)	Std. Error	Sig.[b]	95% Confidence Interval for Difference[b]	
						Lower Bound	Upper Bound
1	1	2	-.218*	.038	<.001	-.302	-.135
	2	1	.218*	.038	<.001	.135	.302
2	1	2	-.301*	.052	<.001	-.416	-.186
	2	1	.301*	.052	<.001	.186	.416
3	1	2	-.243*	.058	.002	-.372	-.114
	2	1	.243*	.058	.002	.114	.372

Based on estimated marginal means

*. The mean difference is significant at the .05 level.

b. Adjustment for multiple comparisons: Bonferroni.

EXPERIMENT 3

PROBABILITY OF RESPONDING CORRECTLY

DURATION (1:SHORT (10S), 2:INTERMEDIATE (12S), 3:LONG (14S)) * STEPS (1:N40, 2:N20, 3:N10)

Pairwise Comparisons

Measure: MEASURE_1

duration	(I) step	(J) step	Mean Difference (I−J)	Std. Error	Sig.[b]	95% Confidence Interval for Difference[b] Lower Bound	Upper Bound
1	1	2	−7.806	3.431	.102	−16.770	1.158
		3	−11.191*	4.258	.048	−22.315	−.067
	2	1	7.806	3.431	.102	−1.158	16.770
		3	−3.385	3.140	.882	−11.588	4.819
	3	1	11.191*	4.258	.048	.067	22.315
		2	3.385	3.140	.882	−4.819	11.588
2	1	2	2.803	5.494	1.000	−11.550	17.156
		3	4.930	3.973	.687	−5.449	15.308
	2	1	−2.803	5.494	1.000	−17.156	11.550
		3	2.126	4.486	1.000	−9.593	13.846
	3	1	−4.930	3.973	.687	−15.308	5.449
		2	−2.126	4.486	1.000	−13.846	9.593
3	1	2	4.280	5.325	1.000	−9.633	18.194
		3	9.083	5.065	.264	−4.150	22.316
	2	1	−4.280	5.325	1.000	−18.194	9.633
		3	4.803	3.806	.664	−5.140	14.746
	3	1	−9.083	5.065	.264	−22.316	4.150
		2	−4.803	3.806	.664	−14.746	5.140

Based on estimated marginal means

*. The mean difference is significant at the .05 level.

b. Adjustment for multiple comparisons: Bonferroni.

ERROR RATE AT 10 SEC.

ERROR (1:E2, 2: E4) * STEP (1:N40, 2:N20, 3:N10)

Pairwise Comparisons

Measure: MEASURE_1

error	(I) step	(J) step	Mean Difference (I−J)	Std. Error	Sig.[b]	95% Confidence Interval for Difference[b] Lower Bound	Upper Bound
1	1	2	.038	.034	.834	−.051	.127
		3	.122*	.036	.009	.027	.217
	2	1	−.038	.034	.834	−.127	.051
		3	.084*	.029	.029	.007	.160
	3	1	−.122*	.036	.009	−.217	−.027
		2	−.084*	.029	.029	−.160	−.007
2	1	2	.018	.027	1.000	−.052	.088
		3	−.016	.022	1.000	−.074	.041
	2	1	−.018	.027	1.000	−.088	.052
		3	−.034	.029	.783	−.110	.043
	3	1	.016	.022	1.000	−.041	.074
		2	.034	.029	.783	−.043	.110

Based on estimated marginal means

*. The mean difference is significant at the .05 level.

b. Adjustment for multiple comparisons: Bonferroni.

Pairwise Comparisons

Measure: MEASURE_1

step	(I) error	(J) error	Mean Difference (I−J)	Std. Error	Sig.[b]	95% Confidence Interval for Difference[b] Lower Bound	Upper Bound
1	1	2	.319*	.044	<.001	.227	.412
	2	1	−.319*	.044	<.001	−.412	−.227
2	1	2	.299*	.039	<.001	.218	.379
	2	1	−.299*	.039	<.001	−.379	−.218
3	1	2	.181*	.042	<.001	.093	.269
	2	1	−.181*	.042	<.001	−.269	−.093

Based on estimated marginal means

*. The mean difference is significant at the .05 level.

b. Adjustment for multiple comparisons: Bonferroni.

ERROR RATE AT 12 SEC.

ERROR (1:-E2, 2: E2) * STEP (1:N40, 2:N20, 3:N10)

Pairwise Comparisons

Measure: MEASURE_1

error	(I) step	(J) step	Mean Difference (I−J)	Std. Error	Sig.[b]	95% Confidence Interval for Difference[b]	
						Lower Bound	Upper Bound
1	1	2	−.050	.041	.713	−.156	.057
		3	−.121*	.038	.014	−.221	−.021
	2	1	.050	.041	.713	−.057	.156
		3	−.071	.035	.169	−.164	.021
	3	1	.121*	.038	.014	.021	.221
		2	.071	.035	.169	−.021	.164
2	1	2	.017	.046	1.000	−.104	.138
		3	.070	.040	.287	−.035	.176
	2	1	−.017	.046	1.000	−.138	.104
		3	.053	.037	.488	−.043	.149
	3	1	−.070	.040	.287	−.176	.035
		2	−.053	.037	.488	−.149	.043

Based on estimated marginal means

*. The mean difference is significant at the .05 level.

b. Adjustment for multiple comparisons: Bonferroni.

Pairwise Comparisons

Measure: MEASURE_1

step	(I) error	(J) error	Mean Difference (I−J)	Std. Error	Sig.[b]	95% Confidence Interval for Difference[b]	
						Lower Bound	Upper Bound
1	1	2	−.026	.072	.726	−.177	.125
	2	1	.026	.072	.726	−.125	.177
2	1	2	.041	.070	.564	−.105	.187
	2	1	−.041	.070	.564	−.187	.105
3	1	2	.166*	.058	.010	.044	.288
	2	1	−.166*	.058	.010	−.288	−.044

Based on estimated marginal means

*. The mean difference is significant at the .05 level.

b. Adjustment for multiple comparisons: Bonferroni.

ERROR RATE AT 14 SEC.

ERROR (1:-E2, 2: -E4) * STEP (1:N40, 2:N20, 3:N10)

Pairwise Comparisons

Measure: MEASURE_1

step	(I) error	(J) error	Mean Difference (I−J)	Std. Error	Sig.[b]	95% Confidence Interval for Difference[b]	
						Lower Bound	Upper Bound
1	1	2	−.261*	.041	<.001	−.347	−.176
	2	1	.261*	.041	<.001	.176	.347
2	1	2	−.271*	.038	<.001	−.351	−.192
	2	1	.271*	.038	<.001	.192	.351
3	1	2	−.346*	.033	<.001	−.414	−.278
	2	1	.346*	.033	<.001	.278	.414

Based on estimated marginal means

*. The mean difference is significant at the .05 level.

b. Adjustment for multiple comparisons: Bonferroni.

EXPERIMENT 4

PROBABILITY OF RESPONDING CORRECTLY

DURATION (1:SHORT (10S), 2:INTERMEDIATE (12S), 3:LONG (14S)) * STEPS (1:N40, 2:N20, 3:N10)

Pairwise Comparisons

Measure: MEASURE_1

step	(I) duration	(J) duration	Mean Difference (I-J)	Std. Error	Sig.[b]	95% Confidence Interval for Difference[b] Lower Bound	Upper Bound
1	1	2	6.008	3.161	.220	-2.333	14.350
		3	13.059	6.872	.221	-5.078	31.197
	2	1	-6.008	3.161	.220	-14.350	2.333
		3	7.051	6.373	.849	-9.769	23.871
	3	1	-13.059	6.872	.221	-31.197	5.078
		2	-7.051	6.373	.849	-23.871	9.769
2	1	2	9.519	4.212	.109	-1.597	20.636
		3	16.206*	6.100	.048	.108	32.305
	2	1	-9.519	4.212	.109	-20.636	1.597
		3	6.687	5.839	.801	-8.723	22.097
	3	1	-16.206*	6.100	.048	-32.305	-.108
		2	-6.687	5.839	.801	-22.097	8.723
3	1	2	8.371	4.229	.190	-2.791	19.532
		3	5.807	6.838	1.000	-12.240	23.853
	2	1	-8.371	4.229	.190	-19.532	2.791
		3	-2.564	5.160	1.000	-16.183	11.056
	3	1	-5.807	6.838	1.000	-23.853	12.240
		2	2.564	5.160	1.000	-11.056	16.183

Based on estimated marginal means

*. The mean difference is significant at the .05 level.

b. Adjustment for multiple comparisons: Bonferroni.

ERROR RATE AT 10 SEC.

ERROR (1:E2, 2: E4) * STEP (1:N40, 2:N20, 3:N10)

Pairwise Comparisons

Measure: MEASURE_1

step	(I) error	(J) error	Mean Difference (I-J)	Std. Error	Sig.[b]	95% Confidence Interval for Difference[b] Lower Bound	Upper Bound
1	1	2	-.013	.068	.849	-.158	.132
	2	1	.013	.068	.849	-.132	.158
2	1	2	.117*	.038	.007	.037	.198
	2	1	-.117*	.038	.007	-.198	-.037
3	1	2	.008	.037	.831	-.072	.088
	2	1	-.008	.037	.831	-.088	.072

Based on estimated marginal means

*. The mean difference is significant at the .05 level.

b. Adjustment for multiple comparisons: Bonferroni.

ERROR RATE AT 14 SEC.

ERROR (1:-E2, 2: -E4) * STEP (1:N40, 2:N20, 3:N10)

Pairwise Comparisons

Measure: MEASURE_1

step	(I) error	(J) error	Mean Difference (I-J)	Std. Error	Sig.[b]	95% Confidence Interval for Difference[b] Lower Bound	Upper Bound
1	1	2	-.317*	.049	<.001	-.421	-.214
	2	1	.318*	.049	<.001	.214	.421
2	1	2	-.354*	.040	<.001	-.441	-.268
	2	1	.354*	.040	<.001	.268	.441
3	1	2	-.252*	.047	<.001	-.352	-.153
	2	1	.252*	.047	<.001	.153	.352

Based on estimated marginal means

*. The mean difference is significant at the .05 level.

b. Adjustment for multiple comparisons: Bonferroni.

EXPERIMENT 1 VS. EXPERIMENT 3

PROBABILITY OF RESPONDING CORRECTLY

GUC (PBAR1: EXPERIMENT 1, PBAR2: EXPERIMENT 3) * DURATION (1:SHORT, 2:INTERMEDIATE, 3:LONG) * STEP (1:N40, 2:N20, 3:N10)

Pairwise Comparisons

Measure: MEASURE_1

duration	step	(I) GUC	(J) GUC	Mean Difference (I−J)	Std. Error	Sig.[b]	95% Confidence Interval for Difference[b] Lower Bound	Upper Bound
1	1	PBAR1	PBAR2	15.195	7.526	.050	−.028	30.417
		PBAR2	PBAR1	−15.195	7.526	.050	−30.417	.028
	2	PBAR1	PBAR2	18.948*	6.387	.005	6.029	31.867
		PBAR2	PBAR1	−18.948*	6.387	.005	−31.867	−6.029
	3	PBAR1	PBAR2	20.430*	6.592	.004	7.096	33.765
		PBAR2	PBAR1	−20.430*	6.592	.004	−33.765	−7.096
2	1	PBAR1	PBAR2	18.370*	5.708	.003	6.825	29.916
		PBAR2	PBAR1	−18.370*	5.708	.003	−29.916	−6.825
	2	PBAR1	PBAR2	22.678*	5.761	<.001	11.025	34.332
		PBAR2	PBAR1	−22.678*	5.761	<.001	−34.332	−11.025
	3	PBAR1	PBAR2	25.564*	5.423	<.001	14.596	36.533
		PBAR2	PBAR1	−25.564*	5.423	<.001	−36.533	−14.596
3	1	PBAR1	PBAR2	25.431*	6.494	<.001	12.296	38.567
		PBAR2	PBAR1	−25.431*	6.494	<.001	−38.567	−12.296
	2	PBAR1	PBAR2	27.338*	6.351	<.001	14.492	40.184
		PBAR2	PBAR1	−27.338*	6.351	<.001	−40.184	−14.492
	3	PBAR1	PBAR2	24.082*	6.208	<.001	11.526	36.638
		PBAR2	PBAR1	−24.082*	6.208	<.001	−36.638	−11.526

Based on estimated marginal means

*. The mean difference is significant at the .05 level.

b. Adjustment for multiple comparisons: Bonferroni.

ERRORS

GUC (PBAR1: EXPERIMENT 1, PBAR2: EXPERIMENT 3) * ERROR (1:E1/E2, 2: E2/E4) * STEP (1:N40, 2:N20, 3:N10)

Pairwise Comparisons

Measure: MEASURE_1

error	step	(I) GUC	(J) GUC	Mean Difference (I−J)	Std. Error	Sig.[b]	95% Confidence Interval for Difference[b] Lower Bound	Upper Bound
1	1	PBAR1	PBAR2	−.106	.055	.060	−.216	.004
		PBAR2	PBAR1	.106	.055	.060	−.004	.216
	2	PBAR1	PBAR2	−.180*	.048	<.001	−.278	−.082
		PBAR2	PBAR1	.180*	.048	<.001	.082	.278
	3	PBAR1	PBAR2	−.132*	.046	.007	−.225	−.039
		PBAR2	PBAR1	.132*	.046	.007	.039	.225
2	1	PBAR1	PBAR2	−.037	.037	.331	−.113	.039
		PBAR2	PBAR1	.037	.037	.331	−.039	.113
	2	PBAR1	PBAR2	−.025	.028	.381	−.083	.032
		PBAR2	PBAR1	.025	.028	.381	−.032	.083
	3	PBAR1	PBAR2	−.069	.036	.063	−.142	.004
		PBAR2	PBAR1	.069	.036	.063	−.004	.142

Based on estimated marginal means

*. The mean difference is significant at the .05 level.

b. Adjustment for multiple comparisons: Least Significant Difference (equivalent to no adjustments).

GUC (PBAR1: EXPERIMENT 1, PBAR2: EXPERIMENT 3) * ERROR (1:-E1/E2, 2: E1/E2) * STEP (1:N40, 2:N20, 3:N10)

Pairwise Comparisons

Measure: MEASURE_1

error	step	(I) GUC	(J) GUC	Mean Difference (I-J)	Std. Error	Sig.[b]	95% Confidence Interval for Difference[b]	
							Lower Bound	Upper Bound
1	1	PBAR1	PBAR2	-.121*	.046	.012	-.215	-.028
		PBAR2	PBAR1	.122*	.046	.012	.028	.215
	2	PBAR1	PBAR2	-.156*	.044	.001	-.244	-.067
		PBAR2	PBAR1	.156*	.044	.001	.067	.244
	3	PBAR1	PBAR2	-.195*	.048	<.001	-.292	-.099
		PBAR2	PBAR1	.195*	.048	<.001	.099	.292
2	1	PBAR1	PBAR2	-.061	.059	.307	-.181	.058
		PBAR2	PBAR1	.061	.059	.307	-.058	.181
	2	PBAR1	PBAR2	-.076	.057	.191	-.190	.039
		PBAR2	PBAR1	.076	.057	.191	-.039	.190
	3	PBAR1	PBAR2	-.060	.042	.167	-.145	.026
		PBAR2	PBAR1	.060	.042	.167	-.026	.145

Based on estimated marginal means

*. The mean difference is significant at the .05 level.

b. Adjustment for multiple comparisons: Bonferroni.

GUC (PBAR1: EXPERIMENT 1, PBAR2: EXPERIMENT 3) * ERROR (1:E1/E2, 2: E2/E4) * STEP (1:N40, 2:N20, 3:N10)

Pairwise Comparisons

Measure: MEASURE_1

error	step	(I) GUC	(J) GUC	Mean Difference (I-J)	Std. Error	Sig.[b]	95% Confidence Interval for Difference[b]	
							Lower Bound	Upper Bound
1	1	PBAR1	PBAR2	-.072*	.032	.031	-.137	-.007
		PBAR2	PBAR1	.072*	.032	.031	.007	.137
	2	PBAR1	PBAR2	-.088*	.028	.003	-.145	-.031
		PBAR2	PBAR1	.088*	.028	.003	.031	.145
	3	PBAR1	PBAR2	-.052	.032	.111	-.116	.012
		PBAR2	PBAR1	.052	.032	.111	-.012	.116
2	1	PBAR1	PBAR2	-.181*	.050	<.001	-.283	-.080
		PBAR2	PBAR1	.181*	.050	<.001	.080	.283
	2	PBAR1	PBAR2	-.187*	.050	<.001	-.289	-.086
		PBAR2	PBAR1	.187*	.050	<.001	.086	.289
	3	PBAR1	PBAR2	-.190*	.046	<.001	-.283	-.098
		PBAR2	PBAR1	.190*	.046	<.001	.098	.283

Based on estimated marginal means

*. The mean difference is significant at the .05 level.

b. Adjustment for multiple comparisons: Bonferroni.

EXPERIMENT 2 VS. EXPERIMENT 4

PROBABILITY OF RESPONDING CORRECTLY

GUC (THROB3: EXPERIMENT 2, THROB4: EXPERIMENT 4) * DURATION (1:SHORT, 2:INTERMEDIATE, 3:LONG) * STEP (1:N40, 2:N20, 3:N10)

Pairwise Comparisons

Measure: MEASURE_1

duration	step	(I) GUC	(J) GUC	Mean Difference (I-J)	Std. Error	Sig.[b]	95% Confidence Interval for Difference[b] Lower Bound	Upper Bound
1	1	THROB3	THROB4	-11.499	6.820	.103	-25.469	2.471
		THROB4	THROB3	11.499	6.820	.103	-2.471	25.469
	2	THROB3	THROB4	.181	7.822	.982	-15.843	16.204
		THROB4	THROB3	-.181	7.822	.982	-16.204	15.843
	3	THROB3	THROB4	8.495	8.246	.312	-8.395	25.386
		THROB4	THROB3	-8.495	8.246	.312	-25.386	8.395
2	1	THROB3	THROB4	-3.309	6.591	.620	-16.809	10.192
		THROB4	THROB3	3.309	6.591	.620	-10.192	16.809
	2	THROB3	THROB4	4.183	5.877	.483	-7.855	16.221
		THROB4	THROB3	-4.183	5.877	.483	-16.221	7.855
	3	THROB3	THROB4	-4.121	7.028	.562	-18.516	10.275
		THROB4	THROB3	4.121	7.028	.562	-10.275	18.516
3	1	THROB3	THROB4	6.407	7.567	.404	-9.093	21.907
		THROB4	THROB3	-6.407	7.567	.404	-21.907	9.093
	2	THROB3	THROB4	-1.537	7.497	.839	-16.893	13.819
		THROB4	THROB3	1.537	7.497	.839	-13.819	16.893
	3	THROB3	THROB4	-14.373*	6.900	.047	-28.508	-.239
		THROB4	THROB3	14.373*	6.900	.047	.239	28.508

Based on estimated marginal means

*. The mean difference is significant at the .05 level.

b. Adjustment for multiple comparisons: Bonferroni.

ERRORS

GUC (THROB3: EXPERIMENT 2, THROB4: EXPERIMENT 4) * ERROR (1:E1/E2, 2: E2/E4) * STEP (1:N40, 2:N20, 3:N10)

Pairwise Comparisons

Measure: MEASURE_1

error	step	(I) GUC	(J) GUC	Mean Difference (I-J)	Std. Error	Sig.[b]	95% Confidence Interval for Difference[b] Lower Bound	Upper Bound
1	1	THROB3	THROB4	.075	.053	.174	-.035	.185
		THROB4	THROB3	-.075	.053	.174	-.185	.035
	2	THROB3	THROB4	-.022	.061	.727	-.148	.105
		THROB4	THROB3	.022	.061	.727	-.105	.148
	3	THROB3	THROB4	-.055	.057	.351	-.173	.064
		THROB4	THROB3	.055	.057	.351	-.064	.173
2	1	THROB3	THROB4	.077*	.029	.015	.017	.138
		THROB4	THROB3	-.077*	.029	.015	-.138	-.017
	2	THROB3	THROB4	.059	.037	.120	-.016	.134
		THROB4	THROB3	-.059	.037	.120	-.134	.016
	3	THROB3	THROB4	.035	.037	.361	-.042	.112
		THROB4	THROB3	-.035	.037	.361	-.112	.042

Based on estimated marginal means

*. The mean difference is significant at the .05 level.

b. Adjustment for multiple comparisons: Bonferroni.

GUC (THROB3: EXPERIMENT 2, THROB4: EXPERIMENT 4) * ERROR (1:-E1/E2, 2: E1/E2) * STEP (1:N40, 2:N20, 3:N10)

Pairwise Comparisons

Measure: MEASURE_1

error	step	(I) GUC	(J) GUC	Mean Difference (I–J)	Std. Error	Sig.[a]	95% Confidence Interval for Difference[a]	
							Lower Bound	Upper Bound
1	1	THROB3	THROB4	.020	.063	.758	-.111	.150
		THROB4	THROB3	-.020	.063	.758	-.150	.111
	2	THROB3	THROB4	.011	.045	.813	-.083	.104
		THROB4	THROB3	-.011	.045	.813	-.104	.083
	3	THROB3	THROB4	.071	.058	.231	-.048	.190
		THROB4	THROB3	-.071	.058	.231	-.190	.048
2	1	THROB3	THROB4	.002	.066	.975	-.133	.138
		THROB4	THROB3	-.002	.066	.975	-.138	.133
	2	THROB3	THROB4	-.001	.040	.985	-.083	.081
		THROB4	THROB3	.001	.040	.985	-.081	.083
	3	THROB3	THROB4	-.007	.050	.886	-.111	.097
		THROB4	THROB3	.007	.050	.886	-.097	.111

Based on estimated marginal means

a. Adjustment for multiple comparisons: Bonferroni.

GUC (THROB3: EXPERIMENT 2, THROB4: EXPERIMENT 4) * ERROR (1:E1/E2, 2: E2/E4) * STEP (1:N40, 2:N20, 3:N10)

Pairwise Comparisons

Measure: MEASURE_1

error	step	(I) GUC	(J) GUC	Mean Difference (I–J)	Std. Error	Sig.[b]	95% Confidence Interval for Difference[b]	
							Lower Bound	Upper Bound
1	1	THROB3	THROB4	-.093	.048	.065	-.192	.006
		THROB4	THROB3	.093	.048	.065	-.006	.192
	2	THROB3	THROB4	-.136*	.046	.006	-.231	-.042
		THROB4	THROB3	.136*	.046	.006	.042	.231
	3	THROB3	THROB4	-.054	.046	.257	-.149	.042
		THROB4	THROB3	.054	.046	.257	-.042	.149
2	1	THROB3	THROB4	.112	.062	.082	-.015	.239
		THROB4	THROB3	-.112	.062	.082	-.239	.015
	2	THROB3	THROB4	.282*	.043	<.001	.193	.371
		THROB4	THROB3	-.282*	.043	<.001	-.371	-.193
	3	THROB3	THROB4	.197*	.056	.002	.082	.313
		THROB4	THROB3	-.197*	.056	.002	-.313	-.082

Based on estimated marginal means

*. The mean difference is significant at the .05 level.

b. Adjustment for multiple comparisons: Bonferroni.

EXPERIMENT 1 VS. EXPERIMENT 2

PROBABILITY OF RESPONDING CORRECTLY

GUC (PBAR: EXPERIMENT 1, THROB: EXPERIMENT 2) * DURATION (1: 3 SEC, 2: 4 SEC, 3: 5 SEC) * STEP (1:N40, 2:N20, 3:N10)

Pairwise Comparisons

Measure: MEASURE_1

duration	step	(I) GUC	(J) GUC	Mean Difference (I-J)	Std. Error	Sig.[b]	95% Confidence Interval for Difference[b] Lower Bound	Upper Bound
1	1	PBAR	THROB	13.577	9.481	.163	-5.814	32.968
		THROB	PBAR	-13.577	9.481	.163	-32.968	5.814
	2	PBAR	THROB	12.056	8.454	.165	-5.235	29.346
		THROB	PBAR	-12.056	8.454	.165	-29.346	5.235
	3	PBAR	THROB	8.944	6.983	.210	-5.336	23.225
		THROB	PBAR	-8.944	6.983	.210	-23.225	5.336
2	1	PBAR	THROB	17.174*	7.744	.035	1.337	33.012
		THROB	PBAR	-17.174*	7.744	.035	-33.012	-1.337
	2	PBAR	THROB	13.297	6.983	.067	-.985	27.579
		THROB	PBAR	-13.297	6.983	.067	-27.579	.985
	3	PBAR	THROB	21.548*	7.900	.011	5.392	37.704
		THROB	PBAR	-21.548*	7.900	.011	-37.704	-5.392
3	1	PBAR	THROB	23.361*	7.312	.003	8.406	38.316
		THROB	PBAR	-23.361*	7.312	.003	-38.316	-8.406
	2	PBAR	THROB	30.678*	7.495	<.001	15.349	46.007
		THROB	PBAR	-30.678*	7.495	<.001	-46.007	-15.349
	3	PBAR	THROB	25.391*	8.592	.006	7.817	42.965
		THROB	PBAR	-25.391*	8.592	.006	-42.965	-7.817

Based on estimated marginal means

*. The mean difference is significant at the .05 level.

b. Adjustment for multiple comparisons: Bonferroni.

ERRORS

GUC (PBAR: EXPERIMENT 1, THROB: EXPERIMENT 2) * ERROR (1:E1/E2, 2: E2/E4) * STEP (1:N40, 2:N20, 3:N10)

Pairwise Comparisons

Measure: MEASURE_1

error	step	(I) GUC	(J) GUC	Mean Difference (I-J)	Std. Error	Sig.[a]	95% Confidence Interval for Difference[a] Lower Bound	Upper Bound
1	1	PBAR	THROB	-.097	.068	.168	-.237	.043
		THROB	PBAR	.097	.068	.168	-.043	.237
	2	PBAR	THROB	-.069	.055	.221	-.181	.044
		THROB	PBAR	.069	.055	.221	-.044	.181
	3	PBAR	THROB	-.059	.046	.205	-.153	.034
		THROB	PBAR	.059	.046	.205	-.034	.153
2	1	PBAR	THROB	-.049	.038	.208	-.128	.029
		THROB	PBAR	.049	.038	.208	-.029	.128
	2	PBAR	THROB	-.065	.041	.123	-.148	.018
		THROB	PBAR	.065	.041	.123	-.018	.148
	3	PBAR	THROB	-.064	.033	.063	-.131	.004
		THROB	PBAR	.064	.033	.063	-.004	.131

Based on estimated marginal means

a. Adjustment for multiple comparisons: Bonferroni.

GUC (PBAR: EXPERIMENT 1, THROB: EXPERIMENT 2) * ERROR (1:-E1/E2, 2: E1/E2) * STEP (1:N40, 2:N20, 3:N10)

Pairwise Comparisons

Measure: MEASURE_1

error	step	(I) GUC	(J) GUC	Mean Difference (I-J)	Std. Error	Sig.[b]	95% Confidence Interval for Difference[b]	
							Lower Bound	Upper Bound
1	1	PBAR	THROB	-.129*	.050	.015	-.231	-.027
		THROB	PBAR	.129*	.050	.015	.027	.231
	2	PBAR	THROB	-.159*	.041	<.001	-.243	-.075
		THROB	PBAR	.159*	.041	<.001	.075	.243
	3	PBAR	THROB	-.135*	.058	.027	-.254	-.016
		THROB	PBAR	.135*	.058	.027	.016	.254
2	1	PBAR	THROB	-.038	.069	.586	-.180	.104
		THROB	PBAR	.038	.069	.586	-.104	.180
	2	PBAR	THROB	.009	.058	.872	-.109	.128
		THROB	PBAR	-.009	.058	.872	-.128	.109
	3	PBAR	THROB	-.079	.054	.152	-.189	.031
		THROB	PBAR	.079	.054	.152	-.031	.189

Based on estimated marginal means

*. The mean difference is significant at the .05 level.

b. Adjustment for multiple comparisons: Bonferroni.

GUC (PBAR: EXPERIMENT 1, THROB: EXPERIMENT 2) * ERROR (1:E1/E2, 2: E2/E4) * STEP (1:N40, 2:N20, 3:N10)

Pairwise Comparisons

Measure: MEASURE_1

error	step	(I) GUC	(J) GUC	Mean Difference (I-J)	Std. Error	Sig.[b]	95% Confidence Interval for Difference[b]	
							Lower Bound	Upper Bound
1	1	PBAR	THROB	-.094*	.028	.002	-.151	-.037
		THROB	PBAR	.094*	.028	.002	.037	.151
	2	PBAR	THROB	-.103*	.034	.006	-.173	-.032
		THROB	PBAR	.103*	.034	.006	.032	.173
	3	PBAR	THROB	-.111*	.043	.015	-.199	-.023
		THROB	PBAR	.111*	.043	.015	.023	.199
2	1	PBAR	THROB	-.160*	.060	.012	-.282	-.037
		THROB	PBAR	.160*	.060	.012	.037	.282
	2	PBAR	THROB	-.231*	.058	<.001	-.349	-.113
		THROB	PBAR	.231*	.058	<.001	.113	.349
	3	PBAR	THROB	-.146*	.062	.026	-.274	-.019
		THROB	PBAR	.146*	.062	.026	.019	.274

Based on estimated marginal means

*. The mean difference is significant at the .05 level.

b. Adjustment for multiple comparisons: Bonferroni.

EXPERIMENT 3 VS. EXPERIMENT 4

PROBABILITY OF RESPONDING CORRECTLY

GUC (PBAR: EXPERIMENT 3, THROB: EXPERIMENT 4) * DURATION (1: 10 SEC, 2: 12 SEC, 3: 14 SEC) * STEP (1:N40, 2:N20, 3:N10)

Pairwise Comparisons

Measure: MEASURE_1

duration	step	(I) GUC	(J) GUC	Mean Difference (I-J)	Std. Error	Sig.[b]	95% Confidence Interval for Difference[b] Lower Bound	Upper Bound
1	1	PBAR	THROB	-13.117*	5.901	.032	-25.064	-1.170
		THROB	PBAR	13.117*	5.901	.032	1.170	25.064
	2	PBAR	THROB	-6.712	5.973	.268	-18.804	5.381
		THROB	PBAR	6.712	5.973	.268	-5.381	18.804
	3	PBAR	THROB	-2.990	7.370	.687	-17.911	11.930
		THROB	PBAR	2.990	7.370	.687	-11.930	17.911
2	1	PBAR	THROB	-4.505	4.913	.365	-14.450	5.441
		THROB	PBAR	4.505	4.913	.365	-5.441	14.450
	2	PBAR	THROB	-5.198	5.127	.317	-15.577	5.181
		THROB	PBAR	5.198	5.127	.317	-5.181	15.577
	3	PBAR	THROB	-8.137	4.746	.095	-17.746	1.472
		THROB	PBAR	8.137	4.746	.095	-1.472	17.746
3	1	PBAR	THROB	4.337	6.700	.521	-9.226	17.900
		THROB	PBAR	-4.337	6.700	.521	-17.900	9.226
	2	PBAR	THROB	1.802	6.398	.780	-11.150	14.754
		THROB	PBAR	-1.802	6.398	.780	-14.754	11.150
	3	PBAR	THROB	-13.064*	5.003	.013	-23.193	-2.936
		THROB	PBAR	13.064*	5.003	.013	2.936	23.193

Based on estimated marginal means

*. The mean difference is significant at the .05 level.

b. Adjustment for multiple comparisons: Bonferroni.

ERRORS

GUC (PBAR: EXPERIMENT 3, THROB: EXPERIMENT 4) * ERROR (1:E1/E2, 2: E2/E4) * STEP (1:N40, 2:N20, 3:N10)

Pairwise Comparisons

Measure: MEASURE_1

error	step	(I) GUC	(J) GUC	Mean Difference (I-J)	Std. Error	Sig.[a]	95% Confidence Interval for Difference[a] Lower Bound	Upper Bound
1	1	PBAR	THROB	.084	.047	.081	-.011	.178
		THROB	PBAR	-.084	.047	.081	-.178	.011
	2	PBAR	THROB	.089	.054	.107	-.020	.199
		THROB	PBAR	-.089	.054	.107	-.199	.020
	3	PBAR	THROB	.018	.055	.743	-.093	.129
		THROB	PBAR	-.018	.055	.743	-.129	.093
2	1	PBAR	THROB	.065	.035	.076	-.007	.137
		THROB	PBAR	-.065	.035	.076	-.137	.007
	2	PBAR	THROB	.020	.025	.443	-.032	.071
		THROB	PBAR	-.020	.025	.443	-.071	.032
	3	PBAR	THROB	.040	.041	.329	-.042	.123
		THROB	PBAR	-.040	.041	.329	-.123	.042

Based on estimated marginal means

a. Adjustment for multiple comparisons: Bonferroni.

GUC (PBAR: EXPERIMENT 3, THROB: EXPERIMENT 4) * ERROR (1:-E1/E2, 2: E1/E2) * STEP (1:N40, 2:N20, 3:N10)

Pairwise Comparisons

Measure: MEASURE_1

error	step	(I) GUC	(J) GUC	Mean Difference (I–J)	Std. Error	Sig.[b]	95% Confidence Interval for Difference[b]	
							Lower Bound	Upper Bound
1	1	PBAR	THROB	.012	.056	.824	-.101	.126
		THROB	PBAR	-.012	.056	.824	-.126	.101
	2	PBAR	THROB	.008	.049	.876	-.092	.107
		THROB	PBAR	-.008	.049	.876	-.107	.092
	3	PBAR	THROB	.131*	.049	.011	.032	.230
		THROB	PBAR	-.131*	.049	.011	-.230	-.032
2	1	PBAR	THROB	.025	.059	.674	-.095	.145
		THROB	PBAR	-.025	.059	.674	-.145	.095
	2	PBAR	THROB	.084	.052	.115	-.021	.190
		THROB	PBAR	-.084	.052	.115	-.190	.021
	3	PBAR	THROB	-.027	.041	.522	-.110	.057
		THROB	PBAR	.027	.041	.522	-.057	.110

Based on estimated marginal means

*. The mean difference is significant at the .05 level.

b. Adjustment for multiple comparisons: Bonferroni.

GUC (PBAR: EXPERIMENT 3, THROB: EXPERIMENT 4) * ERROR (1:E1/E2, 2: E2/E4) * STEP (1:N40, 2:N20, 3:N10)

Pairwise Comparisons

Measure: MEASURE_1

error	step	(I) GUC	(J) GUC	Mean Difference (I–J)	Std. Error	Sig.[b]	95% Confidence Interval for Difference[b]	
							Lower Bound	Upper Bound
1	1	PBAR	THROB	.002	.038	.953	-.074	.079
		THROB	PBAR	-.002	.038	.953	-.079	.074
	2	PBAR	THROB	.030	.030	.326	-.031	.090
		THROB	PBAR	-.030	.030	.326	-.090	.031
	3	PBAR	THROB	.011	.034	.748	-.057	.079
		THROB	PBAR	-.011	.034	.748	-.079	.057
2	1	PBAR	THROB	-.054	.054	.323	-.163	.055
		THROB	PBAR	.054	.054	.323	-.055	.163
	2	PBAR	THROB	-.053	.054	.328	-.162	.056
		THROB	PBAR	.053	.054	.328	-.056	.162
	3	PBAR	THROB	.105*	.042	.018	.019	.190
		THROB	PBAR	-.105*	.042	.018	-.190	-.019

Based on estimated marginal means

*. The mean difference is significant at the .05 level.

b. Adjustment for multiple comparisons: Bonferroni.
